# Supplementary material for: Structural Basis for the Enhanced Infectivity and Immune Evasion of Omicron Subvariants
Source: Viruses. 2023 Jun 20;15(6):1398. doi: 10.3390/v15061398 (PMC10304477; doi:10.3390/v15061398)
Supplement: Supplementary file 1 [file viruses-15-01398-s001.zip › viruses-2422459-supplementary.pdf]

viruses

MDPI

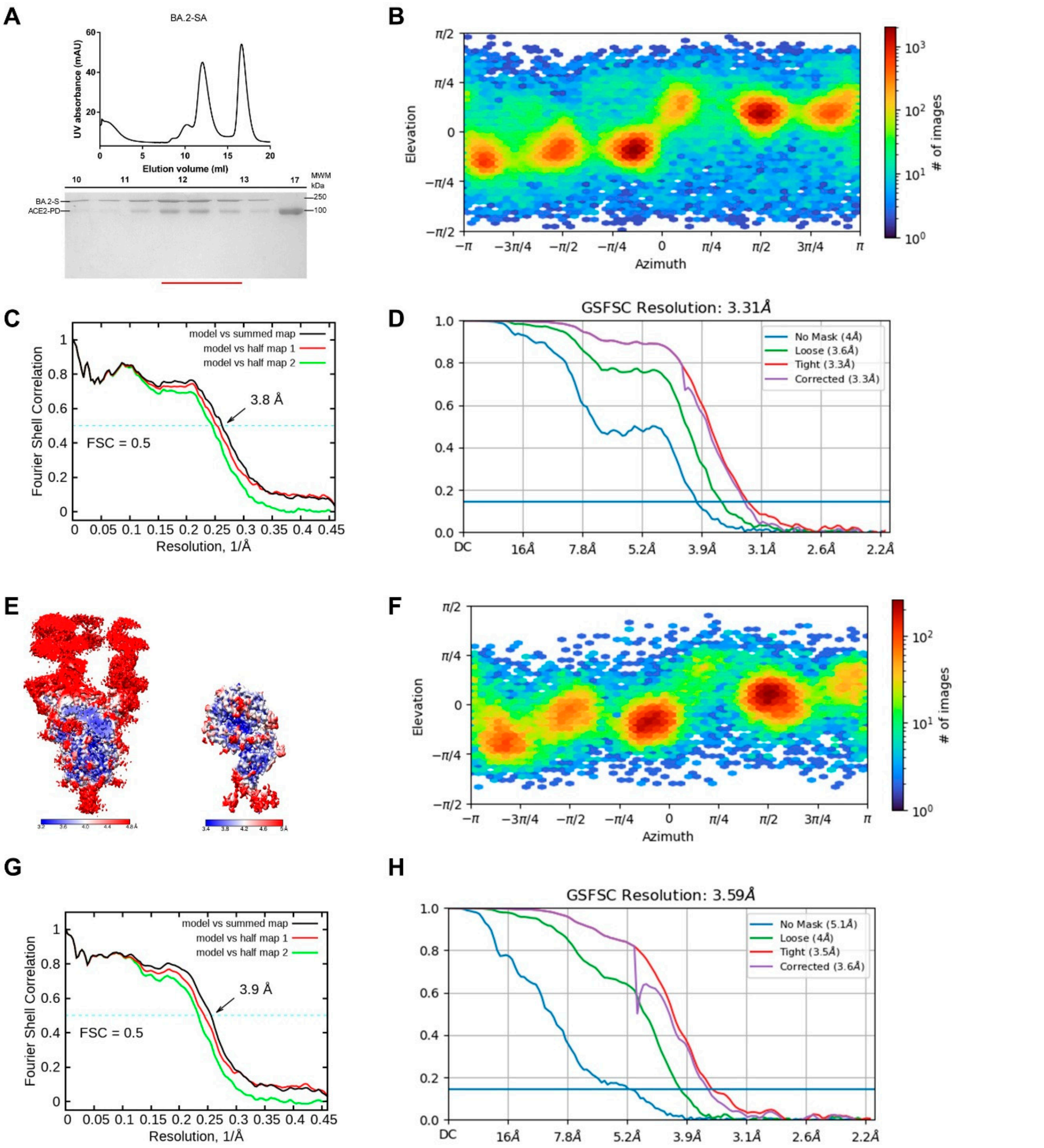

Figure S1. Cryo-EM analysis of S-ECD from BA.2-SA. (A) Representative SEC purification of the BA.2-SA. SDS–PAGE was visualized by Coomassie blue staining and fractions for cryo-EM analysis were marked by red line. **(B)** Euler angle distribution in the final 3D reconstruction of overall map. **(C)** FSC curve of the refined model of BA.2-SA versus the overall structure that it is refined against (black); of the model refined against the first half map versus the same map (red); and of the model refined against the first half map versus the second half map (green). The small difference between the

red and green curves indicates that the refinement of the atomic coordinates did not suffer from overfitting. **(D)** FSC curve of BA.2-SA. **(E)** Local resolution map for the 3D reconstruction of the overall structure and the structure of RBD-PD. **(F)** Euler angle distribution in the final 3D reconstruction of RBD-PD sub-complex. **(G)** FSC curve of the refined model of RBD-PD sub-complex versus the overall structure that it is refined against (black); of the model refined against the first half map versus the same map (red); and of the model refined against the first half map versus the second half map (green). The small difference between the red and green curves indicates that the refinement of the atomic coordinates did not suffer from overfitting. **(H)** FSC curve of RBD-PD sub-complex.

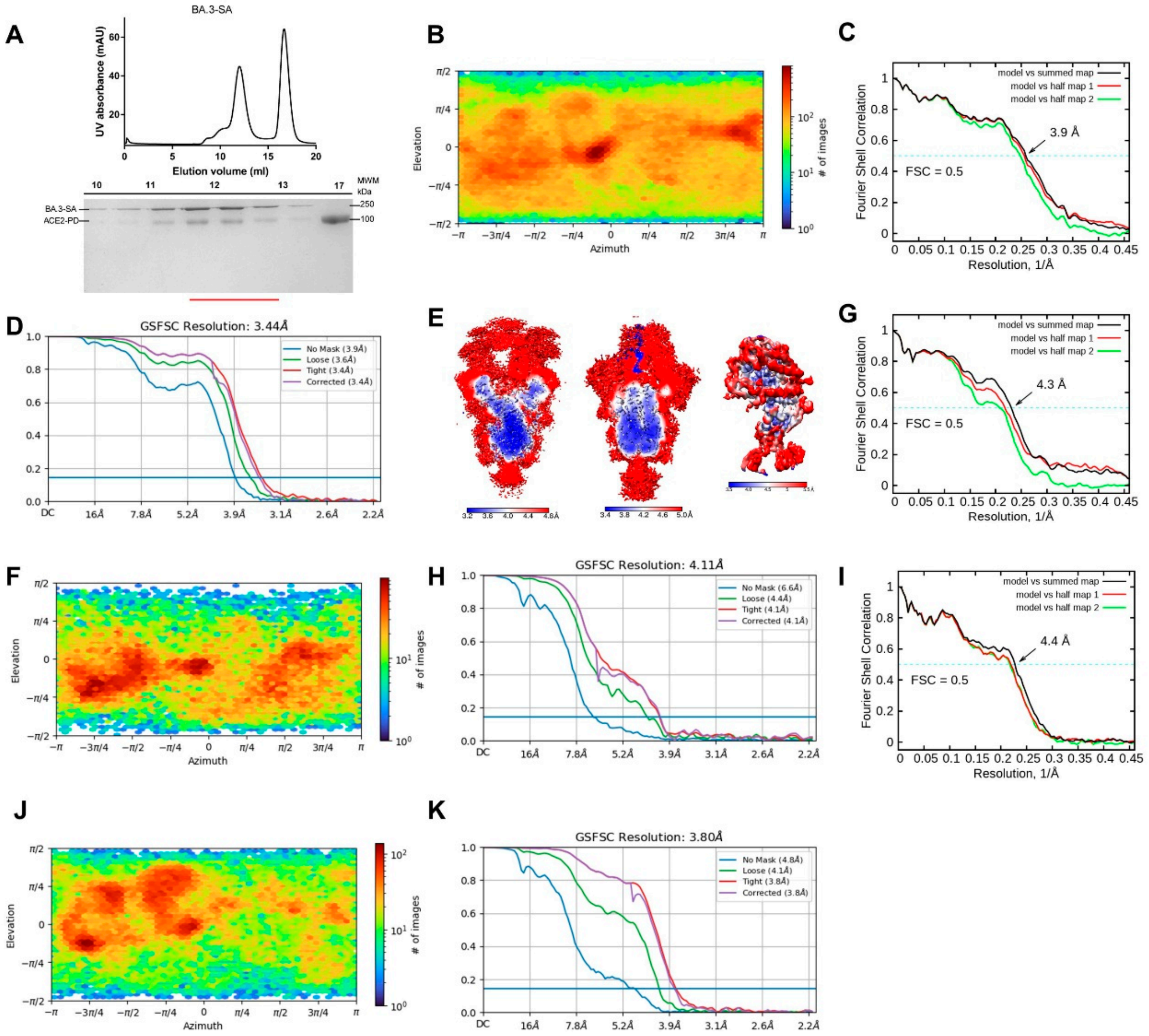

Figure S2. Cryo-EM analysis of BA.3-SA. (A) Representative SEC purification of the BA.3-SA. SDS–PAGE was visualized by Coomassie blue staining and fractions for cryo-EM analysis were marked by red line. **(B)** Euler angle distribution in the final 3D reconstruction of overall map of 3 "up" RBD BA.3-S in complex with 3 PD. **(C)** 3 "up" RBD FSC curve of the refined model of BA.3-S in complex with 3 PD versus the overall structure that it is refined against (black); of the model refined against the first half map versus the same map (red); and of the model refined against the first half map versus the second half map (green). The small difference between the red and green curves indicates that the refinement of the atomic coordinates did not suffer from overfitting. **(D)** FSC curve of 3 "up" BA.3-S in complex with 3 PD. **(E)** Local resolution map for the 3D reconstruction of BA.3-S in complex with PD at 3 "up" RBD 3 PD state (left), 2 "up" RBD 2 PD state (middle) overall structure and RBD-PD sub-complex (right). **(F)** Euler angle distribution in the final 3D reconstruction of RBD-PD sub-complex. **(G)** FSC curve of the refined model of RBD-PD sub-complex versus the overall structure that it is refined against (black); of the model refined against the first half map versus the same map (red); and of the model refined against the first half map versus the second half map (green). The small difference between the red and green curves

indicates that the refinement of the atomic coordinates did not suffer from overfitting. **(H)** FSC curve of RBD-PD sub-complex. **(I)** Euler angle distribution in the final 3D reconstruction of overall map of 2 "up" RBD BA.3-S in complex with 2 PD. **(J)** 2 "up" RBD FSC curve of the refined model of BA.3-S in complex with 2 PD versus the overall structure that it is refined against (black); of the model refined against the first half map versus the same map (red); and of the model refined against the first half map versus the second half map (green). The small difference between the red and green curves indicates that the refinement of the atomic coordinates did not suffer from overfitting. **(K)** FSC curve of 2 "up" RBD BA.3-S in complex with 2 PD.

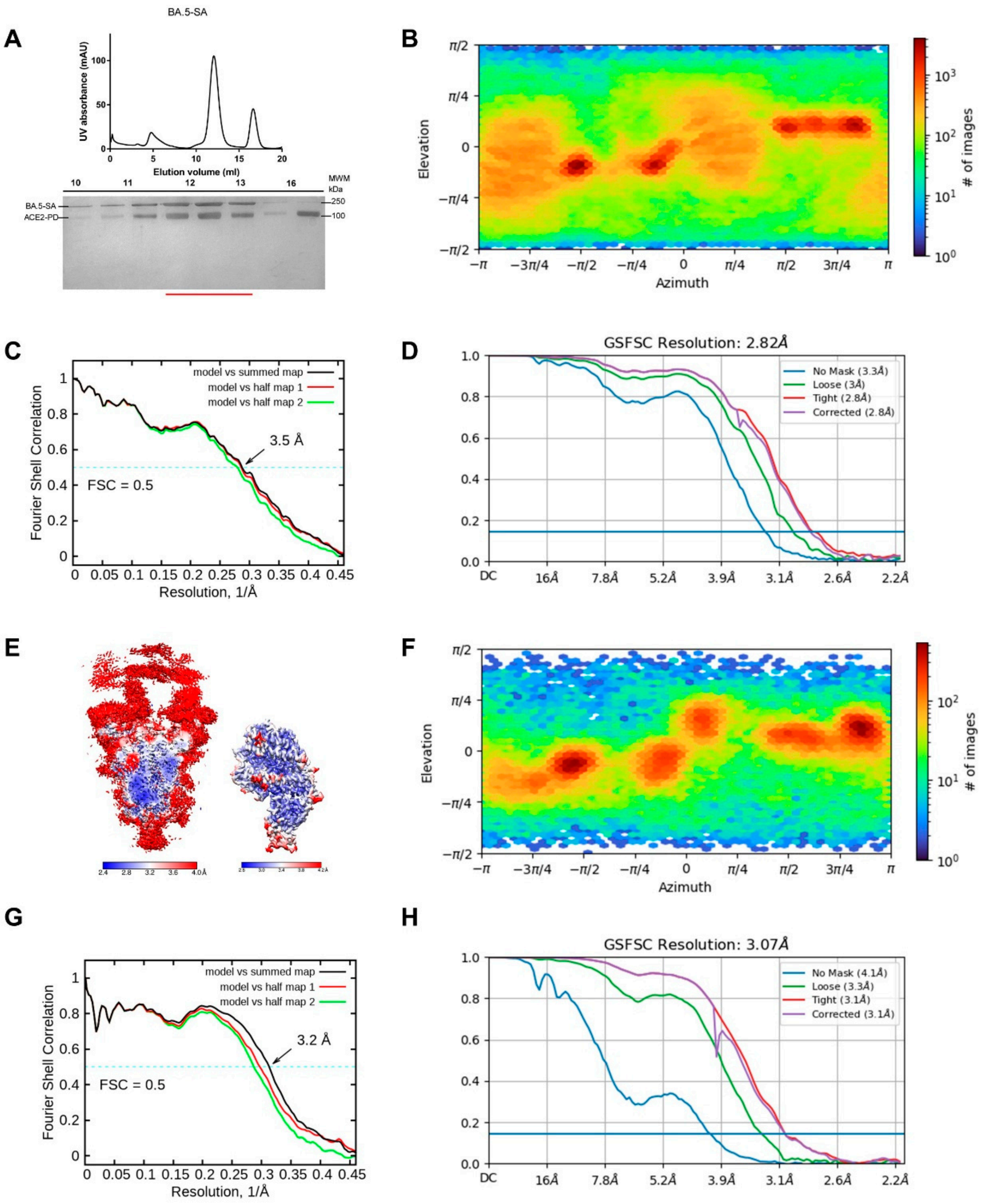

Figure S3. Cryo-EM analysis of S-ECD from BA.5-SA. (A) Representative SEC purification of the BA.5-SA. SDS–PAGE was visualized by Coomassie blue staining and fractions for cryo-EM analysis were marked by red line. **(B)** Euler angle distribution in the final 3D reconstruction of overall map. **(C)** FSC curve of the refined model of BA.5-SA versus the overall structure that it is refined against (black); of the model refined against the first half map versus the same map (red); and of the model refined against the first half map versus the second half map (green). The small difference between the red and green curves indicates that the refinement of the atomic coordinates did not suffer from overfitting. **(D)** FSC

curve of BA.5-SA. **(E)** Local resolution map for the 3D reconstruction of the overall structure and the structure of RBD-PD. **(F)** Euler angle distribution in the final 3D reconstruction of RBD-PD sub-complex. **(G)** FSC curve of the refined model of RBD-PD sub-complex versus the overall structure that it is refined against (black); of the model refined against the first half map versus the same map (red); and of the model refined against the first half map versus the second half map (green). The small difference between the red and green curves indicates that the refinement of the atomic coordinates did not suffer from overfitting. **(H)** FSC curve of RBD-PD sub-complex.

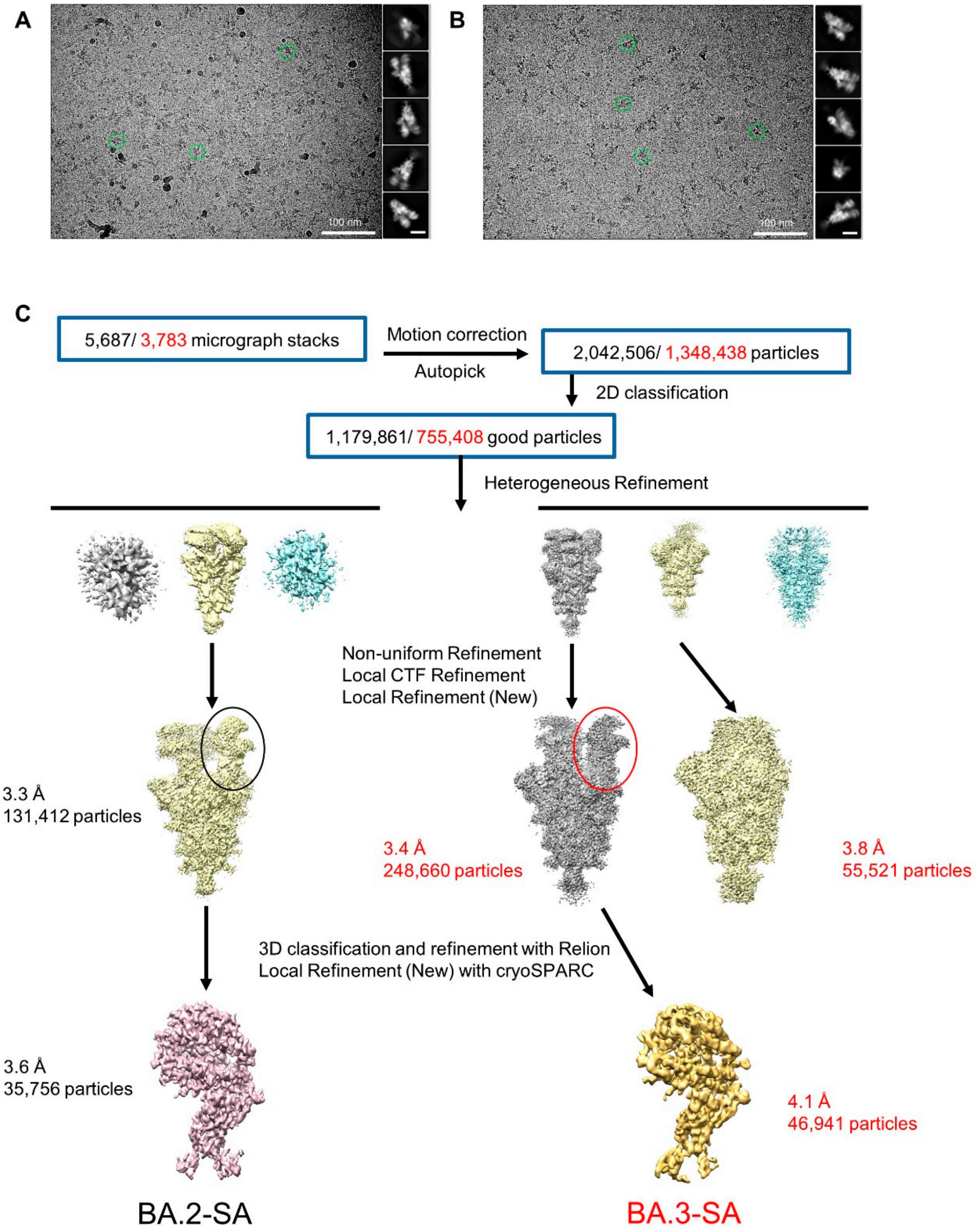

Figure S4. Flowchart of BA.2/3 for cryo-EM data processing. Please refer to the 'Data Processing' in Methods section for details.

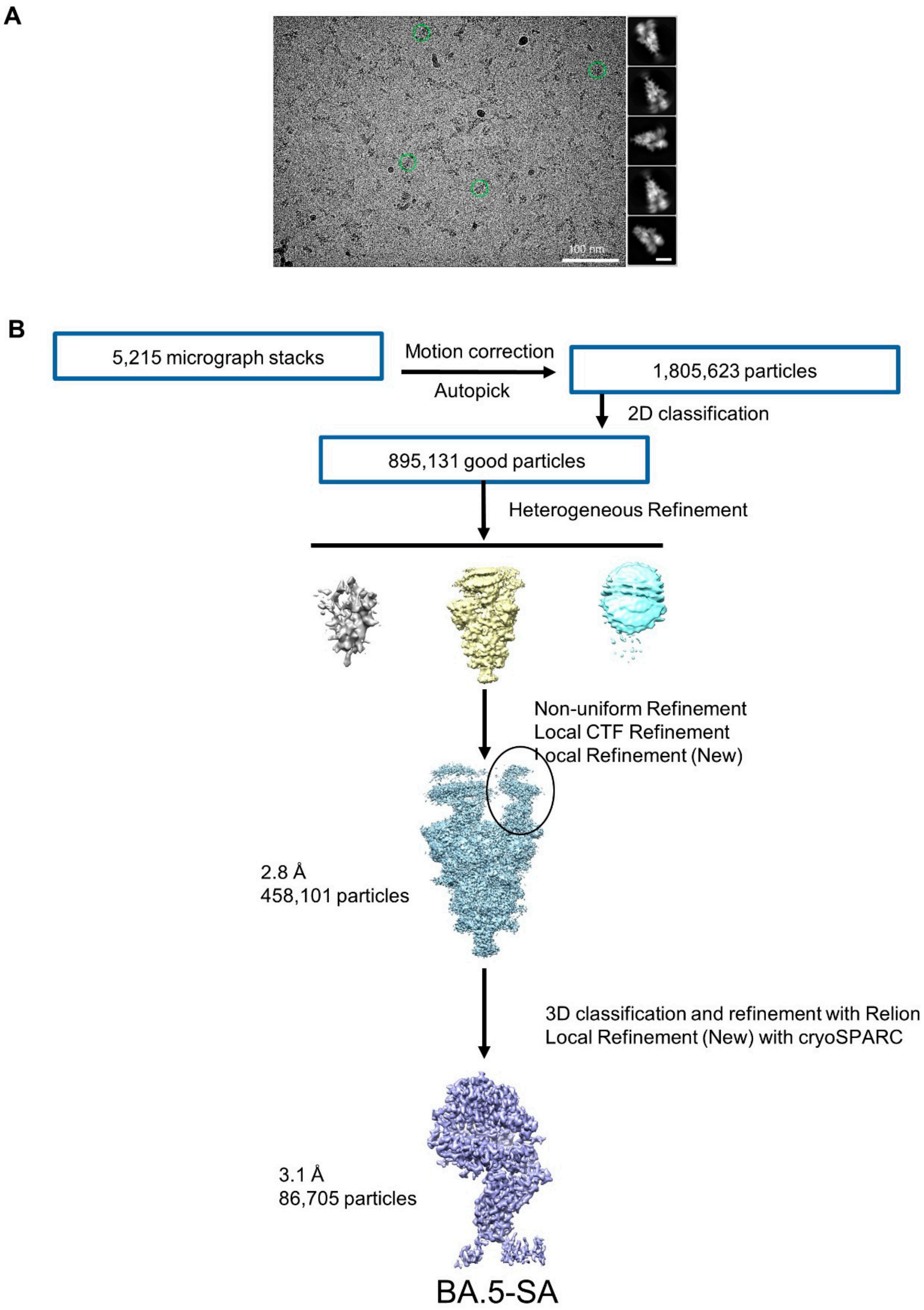

Figure S5. Flowchart of BA.5 for cryo-EM data processing. Please refer to the 'Data Processing' in Methods section for details.

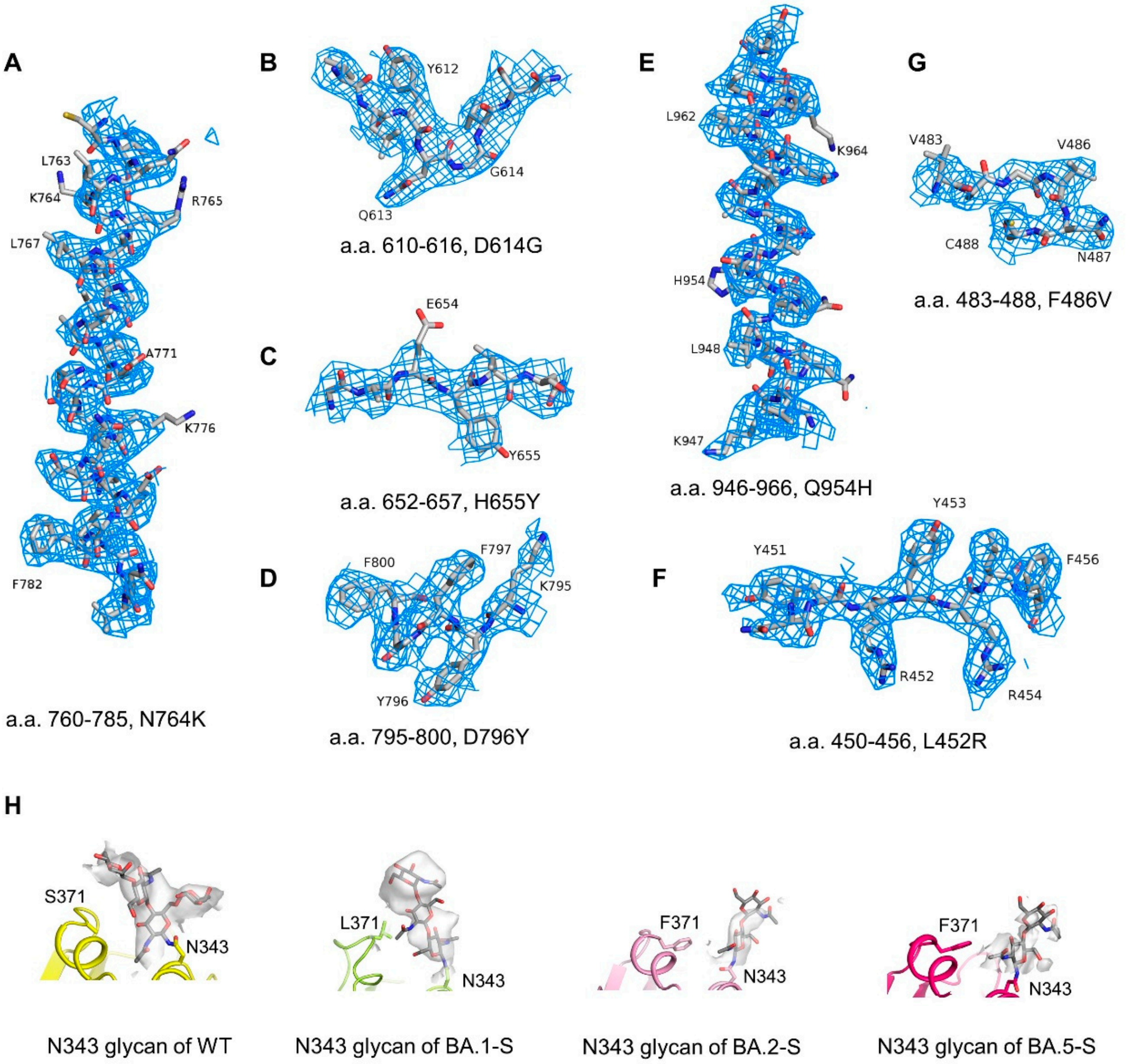

Figure S6. Representative cryo-EM density maps. (A-C) Cryo-EM density map of BA.2-SA is shown at threshold of 7 σ. (**D, E)** Cryo-EM density map of BA.3-SA is shown at threshold of 7 σ. **(F, G)** Cryo-EM density map of BA.5-SA is shown at threshold of 7 σ. **(H)** Cryo-EM density map of N343 glycan from WT spike in complex with S309 (6WPT), BA.1-S, BA.2-S, and BA.5-S is shown at threshold of 8 σ, 10 σ, 5 σ, 4 σ, respectively.

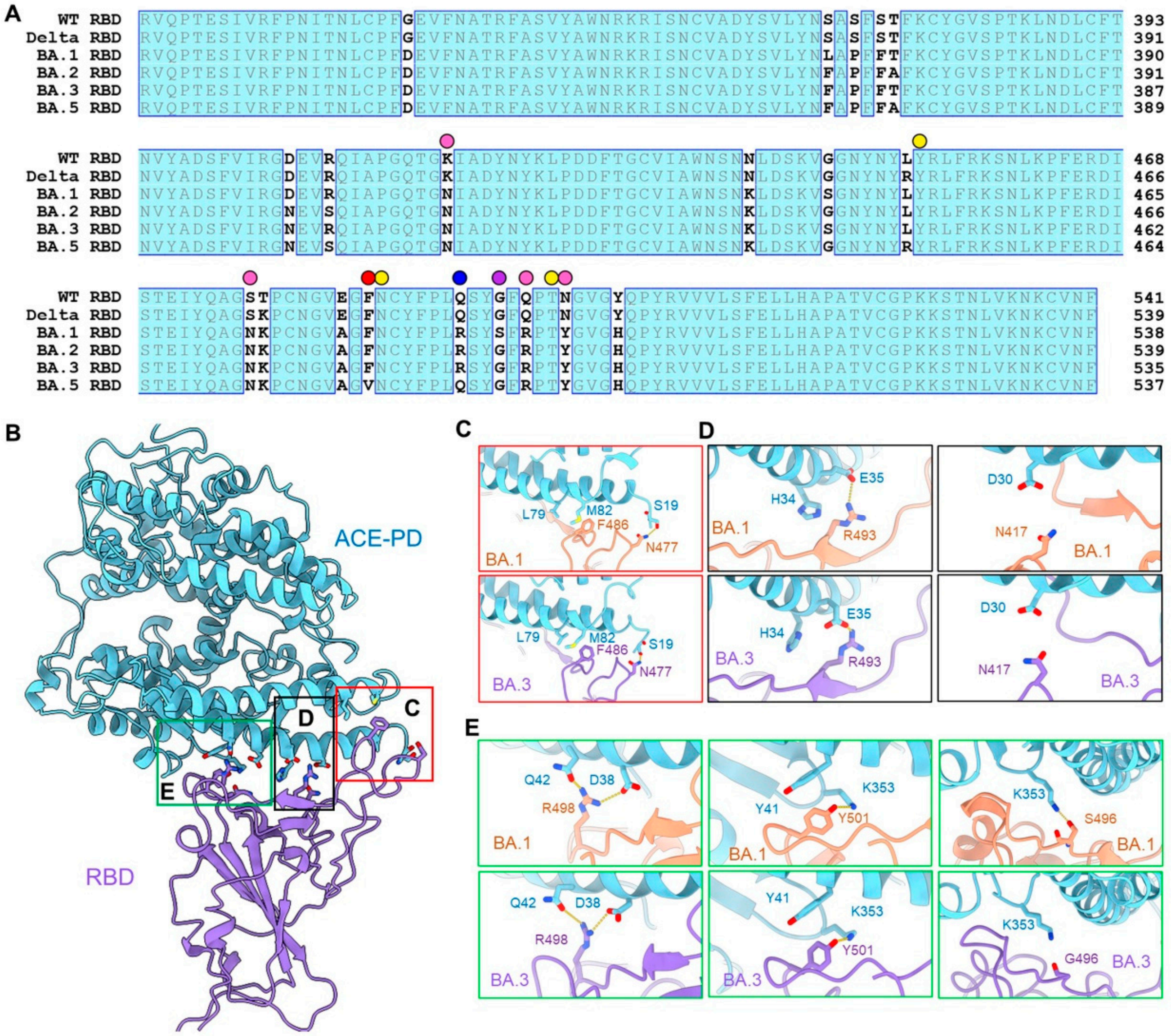

Figure S7. Mutations on the interface of RBD from Omicron sub-lineages and ACE2 underlie their enhanced affinity. **(A)** Sequence alignment of the RBD from indicated SARS-CoV-2 variants. The sequences are aligned using ClustalX. Identical residues are shaded cyan. Residues mapped to the interface with ACE2 are indicated with spheres, yellow for invariant ones and pink for mutated ones. Q493R, indicated by blue sphere, is found in BA.1/2/3. G496S, indicated by purple sphere, is found in BA.1. F486V, indicated by red sphere, is found in BA.5. **(B)** Interaction interfaces between RBD and the PD of ACE2. The boxed regions (from right to left) are shown in **(C), (D)** and **(E)**. **(C)** S477N of RBD in BA.1 and BA.3 forms the H-bond with Ser19 of ACE2. **(D)** Q493R of BA.1 and BA.3 forms new polar interactions with Glu35 of ACE2 via the salt bridges and K417N disrupts the original interaction with Asp30 of ACE2. **(E)** Q498R of BA.1 and BA.3 forms new polar interactions with Asp38 of ACE2 via the salt bridges and N501Y of BA.1 and BA.3 loses the interaction with Tyr41 of ACE2. G496S of RBD in BA.1 sub-lineages form new H-bond with Lys353 of ACE2.

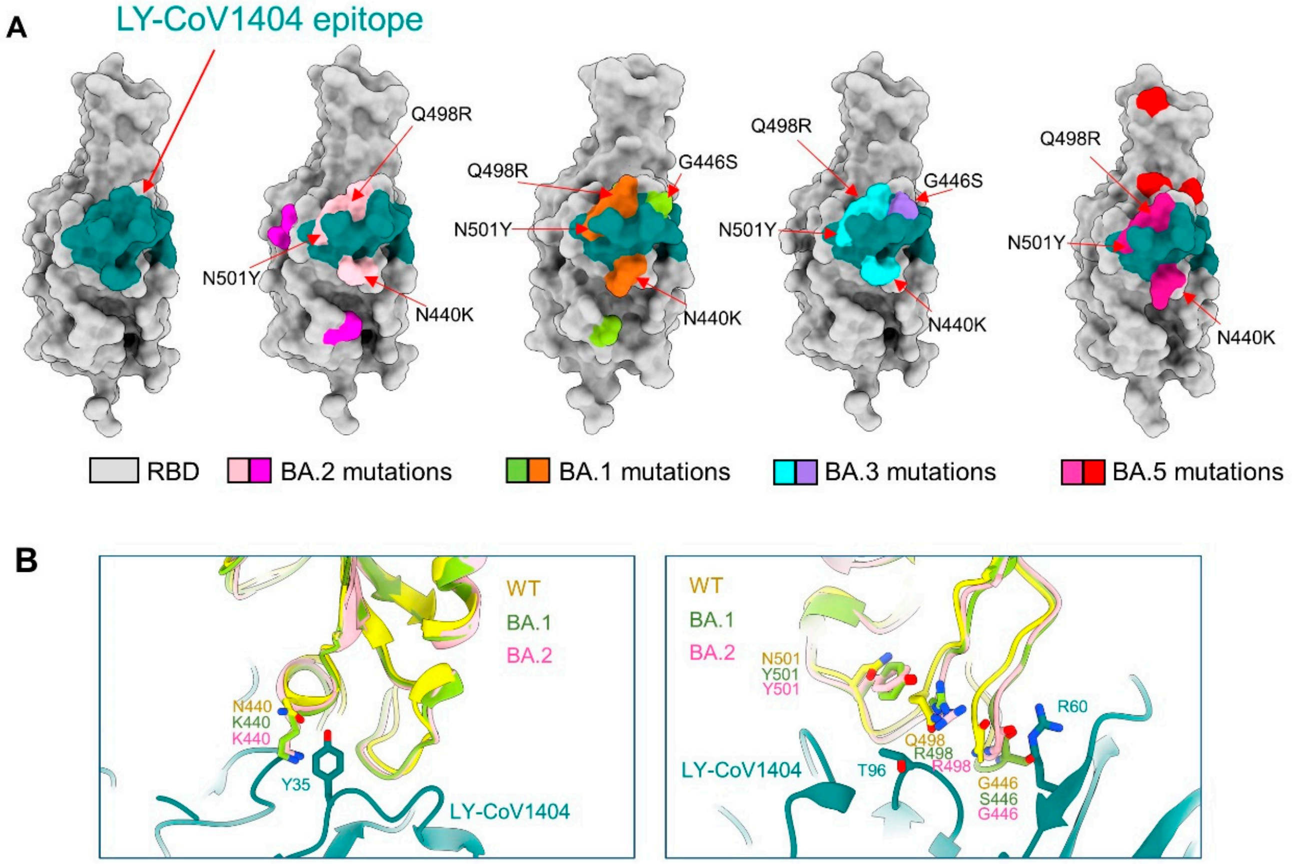

Figure S8. Mapping RBD mutations to representative Class 3 neutralizing antibodies epitopes underlie their neutralizing ability. (A) The binding epitope of antibody LY-CoV1404 bound with the RBD is colored black green (left panel), which overlaps with the mutated residues on BA.2 (middle panel, colored pink), BA.1 (middle panel, colored brown), BA.3 (middle panel, colored cyan), and BA.5 (right panel, colored violet) relative to the RBD (WT) are N440K, G446S, Q498R, and N501Y. The mutations shared only by BA.2 and BA.5 are colored magenta (left panel). The mutated residues on BA.1 (middle panel), BA.3 (middle panel) and BA.5 (right panel) different from BA.2 are colored green, purple and red, respectively. (B) LY-CoV1404 mapped onto the RBD of BA.2-S.

Table S1. Cryo-EM data collection and refinement statistics.

**Data collection**				
EM equipment		Titan Krios (Thermo Fisher Scientific)		
Voltage (kV)		300		
Detector		Gatan K3 Summit		
Energy filter		Gatan GIF Quantum, 20 eV slit		
Pixel size (Å)		1.087		
Electron dose (e-/Å2)		50		
Defocus range (μm)		-1.4 ~ -1.8		
Sample	BA.2-SA	BA.3-SA		BA.5-SA
Number of collected micrographs	5,687	3,783		5,215
**3D Reconstruction**				
Software		cryoSPARC		
Sample	Overall	3 "UP" Overall	2 "UP" Overall	Overall

Number of used particles (Overall)	131,412	248,660	55,521	458,101
Resolution (Å)	3.3	3.4	3.8	2.8
Symmetry		C1		
Map sharpening B-factor (Å2)	109.1	151.2	108.2	111.3
**Refinement**				
Software		Phenix		
Cell dimensions				
a=b=c (Å)		313.056		
α=β=γ (°)		90		
Model composition				
Protein residues	4,786	4,786	4,153	4,786
Side chains assigned	4,786	4,786	4,153	4,786
Sugar	104	104	93	104
R.m.s deviations				
Bonds length (Å)	0.003	0.003	0.005	0.003
Bonds Angle (°)	0.586	0.598	0.661	0.665
Ramachandran plot statistics (%)				
Preferred	93.62	93.45	92.38	93.49
Allowed	6.21	6.21	7.32	6.17
Outlier	0.26	0.34	0.3	0.34
